# Prone positioning for spinal surgery in achondroplasia: A case study report

**DOI:** 10.1177/17504589251359164

**Published:** 2025-07-22

**Authors:** José Miguel Seguro, Pedro Marques, Pedro Moura, Marisa Vicente, José Ferrão

**Affiliations:** 1 Health Sciences Research Unit (UICISA: E), Coimbra Nursing School (ESEnfC), Coimbra, Portugal; 2 Sanfil Medicina, Coimbra, Portugal; 3 Spine Center–Cirurgia da Coluna, Coimbra, Portugal

**Keywords:** Achondroplasia, Case report, Dwarfism, Perioperative care, Prone position, Surgery

## Abstract

**Introduction:**

Prone positioning in surgery, commonly used in orthopaedics and neurosurgery, carries a high risk of positioning-related injuries. In patients with achondroplasia, anatomical differences present unique challenges for safe surgical positioning.

**Case summary:**

This case report describes the perioperative management of a 48-year-old woman with achondroplasia undergoing spinal decompression and posterolateral arthrodesis. A detailed preoperative assessment was conducted, including patient participation in testing positions and equipment to ensure both safety and surgical accessibility. A balanced approach was achieved, and intraoperative positioning was continuously monitored. Postoperative evaluation revealed no positioning-related injuries.

**Conclusions:**

This case highlights the critical role of preoperative planning and intraoperative vigilance in preventing complications. It also underscores the need for specific guidelines addressing the positioning of individuals with achondroplasia during surgery, particularly in the prone position, to be incorporated into international standards and best practice recommendations.

## Introduction

Positioning a surgical patient under general anaesthesia is a complex and high-risk process (Sørensen et al 2015). The goal is to ensure optimal surgical access while protecting the patient from injury (Armstrong & Moore 2022). Once under anaesthesia, the patient is unable to communicate discomfort, making careful planning and assessment crucial (Nascimento & Rodrigues 2020). Of all surgical positions, the prone position carries one of the highest risks of complications, especially to the face, chest, and pressure points such as the knees and genital area (Federico et al 2024, Karahan et al 2022).

When the patient has achondroplasia – a common form of dwarfism – the risks of prone positioning increase significantly. People with achondroplasia often have short limbs, small chest dimensions, limited joint movement, and spinal abnormalities (Kim et al 2021, Whitwell & Fiol 2018). These factors can make standard operating tables and positioning aids unsuitable (Pauli & Botto 2020). Obesity, which is also common in this population, adds to the challenge of increasing pressure on the chest and abdomen, potentially affecting breathing and circulation. Therefore, extra care is needed to support and protect these patients during surgery (Garde-Etayo et al 2024, Saint-Laurent et al 2019).

This case study follows the *CAse Report* (CASE) guidelines for clinical case reporting and highlights the planning, positioning strategies, and outcomes for a patient with achondroplasia undergoing spinal surgery in the prone position (Riley et al 2017).

## Case report

### Patient history and assessment

A 48-year-old woman with achondroplasia presented with worsening lower limb numbness and chronic lower back pain. She lived independently in a home adapted to her needs. Her medical history included scoliosis correction surgery at age 9 and a depressive disorder, currently well-managed with medication.

Preoperative assessment findings:

- Height: 115 cm
- Weight: 58 kg
- BMI: 43.9 kg/m^2^ (classified as morbid obesity)
- Blood pressure: 150/99 mmHg
- Heart rate: 94 bpm
- ECG: Sinus rhythm
- Blood work: Within normal limits

Further observations:

- No varicose veins
- Disproportionate body structure: long torso, short limbs
- Limited shoulder and elbow mobility
- Restricted movement in the neck and lower spine; Thyromental distance – 3 cm
- Modified Mallampati score – Class III (Stutz & Rondeau 2025)
- Low muscle mass and prominent bony areas (increased pressure injury risk)

Spinal imaging (X-ray, computed tomography (CT), and magnetic resonance imaging (MRI)) revealed severe spinal canal narrowing in the lower back and vertebral instability at L4-L5.

The planned procedure was a posterior spinal decompression and fusion from L3 to S1. It was expected to last more than 5 h.

### Preoperative planning

**Figure 1:**
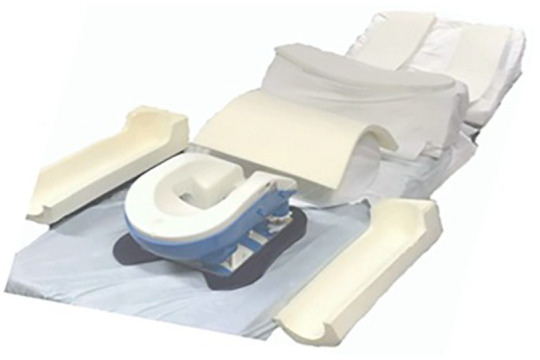
Positioning setup chosen for surgery day

A multidisciplinary team – including surgeons, anaesthetists, and perioperative practitioners – held a preoperative meeting to plan the patient’s positioning and reduce risks (Leong et al 2017, Norton et al 2025). Given the patient’s diagnosis of achondroplasia, personalised assessments and preoperative evaluations were essential to address anatomical variations, potential respiratory compromise, and spinal instability. A simulation of the positioning was undertaken before surgery, with the patient’s input used to identify comfortable and safe alignment (Figure 1).

The final positioning plan included the following:

- Difficult airway anticipated and managed proactively, with preparation of appropriate equipment and technique in advance
- Foam supports under the chest, pelvis, and legs to promote spinal alignment and distribute pressure
- A ProneView® headrest with a mirror to monitor the face during surgery
- A custom arm support setup allowing elbows to rest at 90 ° to prevent nerve compression due to the patient’s limited shoulder mobility

### Intraoperative period

Due to the patient’s obesity, short neck, and limited cervical mobility, features often seen in achondroplasia (Maharjan et al 2024), a difficult airway was anticipated. Acknowledging these challenges, the team took a proactive approach, using a video laryngoscope (C-MAC® with D-Blade®) and a reinforced endotracheal tube, ensuring a safe and uneventful intubation.

Patient positioning was key, as excess adipose tissue in the neck and thorax can impair both movement and visualisation during laryngoscopy. To optimise airway access, the patient was placed in the ‘sniffing’ position, which aligns the oral, pharyngeal, and tracheal axes by flexing the lower cervical spine and extending the atlanto-occipital joint.

**Figure 2:**
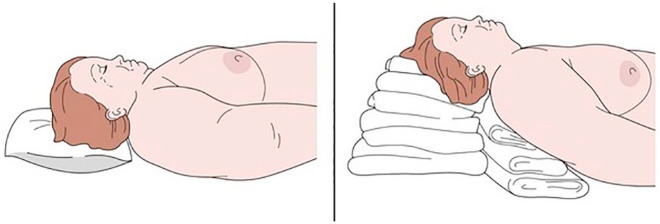
Positioning for intubation in an obese patient with short neck and anticipated difficult airway (Wiener-Kronish & Shimabukuro 2008)

Figure 2 illustrates the anatomical challenges commonly encountered in obese patients with a short neck, as observed in this case. It also depicts the airway management approach adopted, including patient positioning and equipment choice, highlighting the adaptations required to ensure a safe and effective intubation. The presence of thoracic kyphosis further complicated the scenario, reinforcing the need for meticulous preoperative planning and close multidisciplinary collaboration.

**Figure 3:**
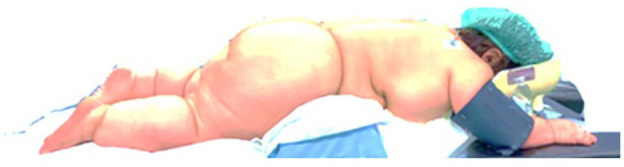
Positioning setup chosen for surgery (before the warm blanket was placed)

After anaesthesia induction, the patient was turned into the prone position as planned. Although custom foam arm supports were trialled during the preoperative consultation, a reassessment on the day of surgery indicated that the additional foam support was not required. Instead, radiolucent arm boards were used, providing adequate positioning while allowing for improved access during intraoperative imaging (Figure 3).

Five team members helped coordinate the movement for safety. The procedure lasted 5 h 20 min with no complications recorded.

Intraoperative care included the following:

- Pressure point checks every 30 min (face, shoulders, elbows, knees, feet)
- Regular reassessment of chest foam positioning to avoid tracheal compression, particularly important given the patient’s short neck and altered upper body anatomy
- Continuous observation of facial skin via the headrest mirror
- Maintenance of normothermia using a forced-air warming blanket
- Monitoring blood glucose every 2 h to ensure stability
- Temperature kept at 37 °C; only surgical areas were uncovered

### Guidelines and best practices

Perioperative care was based on *Association of periOperative Registered Nurses* (AORN) guidelines on patient positioning (2024); *European Pressure Ulcer Advisory Panel* (EPUAP)/*National Pressure Injury Advisory Panel* (NPIAP)/*Pan Pacific Pressure Injury Alliance* (PPPIA) recommendations for pressure injury prevention in surgical settings (2019) and *Pressure Ulcers Prevention on PROne Position* (PUPRO) (Ramos et al 2020).

Structured briefing prior to anaesthesia and debriefing after surgery were conducted to enhance team communication, anticipate risks, and ensure safe patient management (Leong et al 2017, Norton et al 2025). The positioning plan was reviewed during the surgical safety briefing prior to anaesthetic induction, in accordance with ‘Spine Center – Cirurgia da Coluna’ intraoperative nursing care protocols.

### Postoperative evaluation

At the end of surgery, the patient was safely repositioned supine and extubated without issue. The patient was transferred to the Post-Anaesthesia Care Unit without requiring additional airway support.

Postoperative assessments showed the following:

- No signs of pressure injury or nerve damage
- Intact facial skin
- Effective pain control
- Mobilisation began on the second postoperative day
- Discharge occurred on the sixth day with no complications

## Discussion

Prone positioning has improved over time, thanks to better materials and equipment. However, for patients with achondroplasia, additional steps are necessary to tailor care (Souza et al 2023). Their unique body structure, including limited joint range and spinal abnormalities, requires custom supports and careful assessment (Pauli 2019). Preoperative simulations with patient participation help ensure safety and comfort. This dialogue strengthens trust and supports shared decision-making (Gonçalves et al 2024).

Throughout the procedure, regular skin checks and temperature control were essential. The ProneView headrest allowed continuous visual checks of the face, adding an extra layer of safety.

Airway management was another key concern. Patients with achondroplasia are at higher risk for difficult intubation due to skeletal features and concomitant comorbidities (such as obesity). Planning and using video-assisted tools helped avoid complications in this case. Use of custom foam supports, such as double cushioning beneath the chest and pelvis, not only improves alignment but also helps prevent pressure injuries during lengthy procedures (Oliveira et al 2018). Regular reassessment during surgery is essential to ensure skin integrity and adequate tissue perfusion. Recommendations such as supporting the chest and pelvis to allow free abdominal movement, keeping the head at or above heart level, and slightly tilting the table in a reverse Trendelenburg position help minimise intraocular pressure and venous stasis (Cunha et al 2023).

The absence of complications, such as pressure injuries, nerve damage, or visual loss, shows how effective a team-based, patient-specific approach can be (American Society of Anesthesiologists Task Force on Perioperative Visual Loss et al 2019, He et al 2024, Roth et al 2019). Postoperative skin assessments and early mobilisation also played an important role in recovery (Souza et al 2023).

Despite these positive outcomes, there are still no specific surgical positioning guidelines for patients with achondroplasia. Including these individuals in perioperative protocols is essential for safe and inclusive care.

## Conclusion and recommendations

This case highlights the importance of customised surgical positioning in patients with achondroplasia. Their anatomical and physiological differences require careful preoperative assessment, adapted positioning equipment, and close teamwork between surgical, anaesthetic, and nursing teams or perioperative practitioners.

Using tailored supports and planning strategies helped avoid complications and ensured a safe outcome. However, current guidelines often overlook the specific needs of patients with this skeletal dysplasia. Future updates to perioperative care standards should include evidence-based recommendations for this population to promote equity and safety in surgical care.
